# Computer-aided efficient design and performance optimization of cutting head for roadheader

**DOI:** 10.1038/s41598-022-10702-1

**Published:** 2022-04-26

**Authors:** Xin Jin, Guochao Zhao, Lijuan Zhao, Guocong Lin

**Affiliations:** School of Mechanical Engineering, Liaoning Technical University, Fuxin, 123000 China

**Keywords:** Engineering, Mathematics and computing

## Abstract

The cutting head is the core working mechanism of the roadheader for coal-rock materials cutting. The efficient and high performance design of cutting head is the key to improve the road head digging and mining technology. In this paper, based on cutting head design theory and virtual prototype technology, we propose a computer-aided structure design and performance optimization method for cutting head. We compile the calculation code and realize the reading and storing of relevant data through Excel. In particular, to obtain more realistic cutting performance data of the cutting head, we construct a coupling model of cutting head cutting rock wall based on virtual prototype technology, and then establish a database matching structural parameters, working parameters, coal-rock properties and cutting performance through extensive simulations. Based on the method, we complete the design of EBZ220 roadheader cutting head. We show that our method can realize the fast and efficient design of cutting head, and the designed cutting head has good working performance.

## Introduction

The roadheader is mainly composed of traveling mechanism, working mechanism, loading mechanism and reloading mechanism. In the process of tunneling and coal mining, the cutting head constantly crushes the coal-rock materials, which is the core working mechanism of the roadheader. Therefore, the efficient and reasonable design of the cutting head is the key to ensure the cutting performance and service life of the roadheader^1, 2^. Since as early as the 1960s, a lot of research has been carried out on the design and performance analysis of the cutting head of roadheader. Hekimoglu^3, 4^ conduct a comparative analysis of the stresses on two types of roadheaders, longitudinal and transverse axis, and demonstrate that the equal circumferential spacing arrangement of the picks is an important factor to ensure the stability of the cutting head. Anon^5^ develop a model for predicting the performance of a roadheader cutting hard rock based on experimental tests on rock samples. Acaroglu et al.^6^ develop a software for stability analysis of roadheader and analyze the effect of pick inclination and cutting head shape on the stability. Ebrahimabadi et al.^7, 8^ investigate the effect of rock brittleness index on the performance of roadheader and establish a new empirical formula to predict the performance of roadheader under different cutting conditions. Moreover, they develop a database containing transient cutting rates and parameters of coal-rock mechanical properties, and analyzed the database using statistical methods to obtain a prediction model for cutting performance of roadheader. Li and Dong^9, 10^ simulate the cutting process of roadheader by finite element method, and analyze the stress of pick and coal-rock material. Dogruoz et al.^11^ investigate the wear dullness of picks cutting rocks of different hardness developed a numerical model between the energy consumed per unit volume and the type of picks, wear conditions, and mechanical properties of the rock. Wang et al.^12^ study the load fluctuation of cutting head of longitudinal axis roadheader by mathematical method and find that the frequency of low frequency fluctuation is the product of spiral number and rotation frequency, while the frequency of high frequency fluctuation is related to the circumferential spacing of cutting picks. Zhang et al.^13^ study the influence of the number of spirals on the performance of the cutting head and find that compared with the two-spiral cutting head, the three-spiral cutting head has a larger average cutting area, less load fluctuation and less variation of the effective cutting picks. Huang et al.^14^ propose a genetic algorithm-based cutoff spacing optimization design method with the goal of minimizing load fluctuation. Li^15^ propose a design scheme for variable angle spiral line arrangement of picks based on TRIZ theory to improve the cutting performance of cutting head, and carry out experimental verification. Yuan^16, 17^ et al. explore the optimal interval of cutting angle and rotation angle of picks. Liu et al.^18^ summarize the research trends of cutting head design analysis and propose that future research presents the characteristics of mutual verification of theory and experiment, two-way coupling of finite element and discrete element, and combination of intelligent algorithm and virtual simulation. Piotr Cheluszka^19^ simulated cutting of rocks with different uniaxial compressive strengths (UCS) in automatic and manual mode and the behaviour of the roadheader during the cutting of rocks with variable workability was studied. It was found that the algorithm developed for automatic control of the cutting heads’ movement allows reducing the consumption of cutting energy by up to half compared to the consumption during cutting in manual mode, while also improving the dynamic state of the machine.

With the development of computer technology, the first cutting head design software package for roadheader appeared in the 1980s, And then, scholars began to apply it to the cutting head design process continuously^20, 21^. By the 1990s, many scholars have developed a variety of software packages for cutting head design. Some of these software packages can generate cutting diagrams and calculate forces and moments of picks; some can obtain information on power, moments and circumferential forces under different cutting circumference angles; some can generate pick arrangement diagrams and draw curves of load and cutting specific energy consumption^22–28^. At the early twenty-first century, Tiryaki et al.^29^ develop a new software for the design of cutting head for roadheader, which takes into account the equation of cutting force coefficient in the prediction of force on picks compared with the previous auxiliary design software. The software can reflect the difference of force and chip between one pick per line and three picks per line. However, the software does not consider the relationship between rock mechanical properties and the prediction of cutting specific energy consumption and the selection of cutting head design. Later, Li^30–33^ compile a program to simulate the chip diagram of picks under the drilling condition of roadheader. In addition, He and other scholars^34, 35^ jointly prepare a design program for spiral line and pick distribution of cutter head and a program to compare the force and chip shape of cutting head under different picks arrangement. Qin^36^ develop an interactive parametric design system for cutting heads based on ANSYS APDL and VC +  + language, which greatly improved the design efficiency and design quality. Wei^37^ develop a longitudinal shaft roadheader dynamic stability analysis software. Piotr Cheluszka^38^ propose a computer-aided design of working units of mining machines, and the automation of manufacturing process of the working units of mining machines is realized by offline programming. A few years later, Piotr Cheluszka^39^ develop a mathematical measurement model and procedures that allow automatic positioning of the camera system to the photographed objects, as well as acquisition and analysis of the measurement images.

The design software of cutting head for roadheader is constantly innovated and improved, and the software functions and factors considered are more comprehensive. Based on the above research results, the computer-aided efficient design and performance optimization of cutting head is proposed in this study, which visualizes the cutting head design process by compiling calculation code, and reading and storing the relevant data in combination with Excel. It is worth mentioning that the cutting performance in the proposed approach is not completely calculated by theoretical equations, but based on the virtual prototype technology, the coupling model of cutting head cutting rock wall is constructed, and a database matching the structural parameters, working parameters, coal-rock properties and cutting performance is established through a large number of simulations. Through the design and application of EBZ220 cutting head, it is found that the design process is convenient and fast, and the cutting head has good performance, which indicates that the proposed approach can provide a clear quantitative basis for its structural design and performance optimization.

## Kinematic theory of cutting head

**Figure 1:**
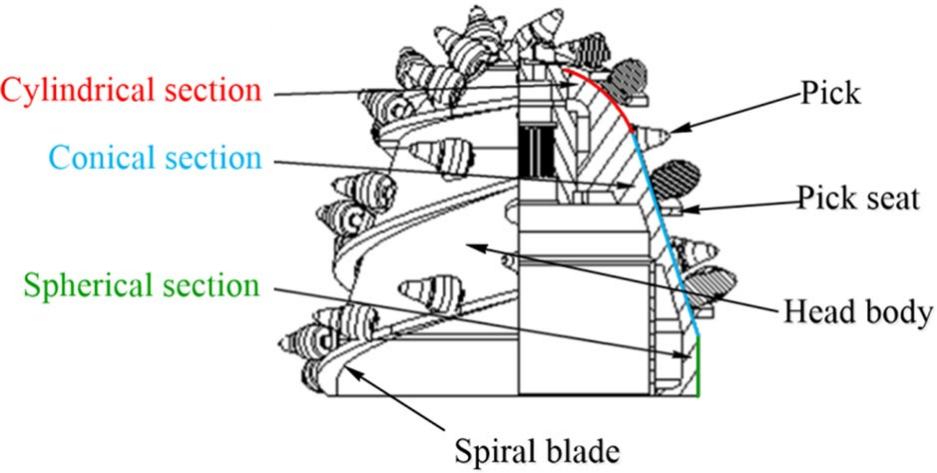
Structure composition of cutting head.

The cutting head is composed of head body, helical vane, pick and pick holder, as shown in Fig. 1. The helical vane is welded to the head body in the form of segmental rolling or sheet metal lap, and the pick is installed in the pick holder and welded in the direction of the helical vane according to the intercept distance. The shape of cutting head usually consists of cylindrical section, conical section and spherical section, and its structural parameters include cutting head length, diameter, isometric principle angle, spiral angle, etc.

**Figure 2:**
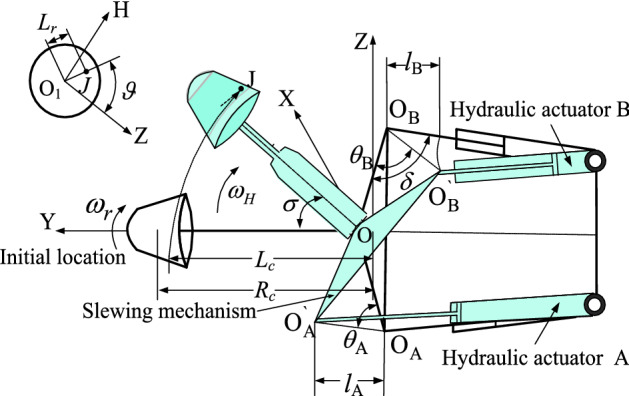
Spatial position of the cutting head and the hydraulic actuator.

In the process of cross-swing cutting, the cutting head not only rotates around its own axis, but also swings with the cantilever, and the spatial position of the cutting head and the hydraulic actuator is shown in Fig. 2. In which, the X-axis is defined as the vertical working surface direction, the Y-axis is the cutting depth direction, the cutting head is swung reciprocally in the YOZ plane and HO_1_Z is the local coordinate system for the large end face of the cutting head.

As shown in Fig. 2, the hydraulic actuators A and B are arranged symmetrically, and the initial lengths are *l*. The distance from the extension of the hydraulic actuators to the center of the slewing mechanism is equal, i.e. $\left| {{\text{O}}_{{\text{A}}} {\text{O}}} \right| = \left| {{\text{O}}_{{\text{B}}} {\text{O}}} \right|$. The angle between the hydraulic actuator and the center of the slewing mechanism is equal, noted as $\delta$. The cross-swing radius of the cutting head is *R*_*c*_. The relationship between the cross-swing angle $\sigma$ and the cross-swing speed *v* can be expressed as follows.

1 $$\sigma = \frac{vT}{{R_{c} }}$$

where *v* is cross-swing speed, mm/s; *T* is one-way cross-swing time, s.

At this moment, the expansion and contraction of the hydraulic actuator are *l*_A_, *l*_B_, and the extensions are noted as ${\text{O}}_{{\text{A}}}^{\prime}$, ${\text{O}}_{{\text{B}}}^{\prime}$. According to the geometric and kinematic relations, the following system of equations is obtained.

2 $$\left\{ {\begin{array}{*{20}c} {\left| {{\text{O}}_{{\text{A}}} {\text{O}}_{{\text{A}}}^{\prime} } \right|^{2} + l^{2} - 2l\left| {{\text{O}}_{{\text{A}}} {\text{O}}_{{\text{A}}}^{\prime} } \right|\cos (\delta + \theta_{{\text{A}}} ) = (l + l_{{\text{A}}} )^{2} } \\ {\left| {{\text{O}}_{{\text{A}}} {\text{O}}_{{\text{A}}}^{\prime} } \right|^{2} = \left| {{\text{OO}}_{{\text{A}}} } \right|^{2} + \left| {{\text{OO}}_{{\text{A}}}^{\prime} } \right|^{2} - 2\left| {{\text{OO}}_{{\text{A}}} } \right|\left| {{\text{OO}}_{{\text{A}}}^{\prime} } \right|\cos \sigma } \\ {\theta_{{\text{A}}} = (180^{ \circ } - \sigma )/2} \\ \end{array} } \right.$$

3 $$\left\{ {\begin{array}{*{20}c} {\left| {{\text{O}}_{{\text{B}}} {\text{O}}_{{\text{B}}}^{\prime} } \right|^{2} + l^{2} - 2l\left| {{\text{O}}_{{\text{B}}} {\text{O}}_{{\text{B}}}^{\prime} } \right|\cos (\delta - \theta_{{\text{B}}} ) = (l - l_{{\text{B}}} )^{2} } \\ {\left| {{\text{O}}_{{\text{B}}} {\text{O}}_{{\text{B}}}^{\prime} } \right|^{2} = \left| {{\text{OO}}_{{\text{B}}} } \right|^{2} + \left| {{\text{OO}}_{{\text{B}}}^{\prime} } \right|^{2} - 2\left| {{\text{OO}}_{{\text{B}}} } \right|\left| {{\text{OO}}_{{\text{B}}}^{\prime} } \right|\cos \sigma } \\ {\theta_{{\text{B}}} = (180^{ \circ } - \sigma )/2} \\ \end{array} } \right.$$

Therefore, the relationship between expansion, contraction and cross-swing angle can be obtained from Eqs. (2)~(3):

4 $$l_{A} = \sqrt {2a^{2} (1 - \cos \sigma ) + l^{2} + 2al\sqrt {2(1 - \cos \sigma )} \cdot \sin (\delta - \frac{\sigma }{2})} - l$$

5 $$l_{B} = \sqrt {2a^{2} (1 - \cos \sigma ) + l^{2} - 2al\sqrt {2(1 - \cos \sigma )} \cdot \sin (\delta - \frac{\sigma }{2})}$$

where *a* is the distance from the centre of the rotary table to the rotary centre of the hydraulic actuator, mm.

The distance from the pick J to the swing center of the cantilever is recorded as *L*_*c*_, and the distance to the axis of the cutting head is recorded as *L*_*r*_. Therefore, The equation of motion of the pick tip is derived as follows:

6 $$\left\{ {\begin{array}{*{20}c} {x = L_{c} \sin (\omega_{H} T) + L_{r} \sin (\omega_{r} T)\sin (\omega_{H} T)} \\ {y = L_{c} \cos (\omega_{H} T) - L_{r} \sin (\omega_{r} T)\sin (\omega_{H} T)} \\ {z = L_{r} \cos (\omega_{r} T)} \\ \end{array} } \right.$$

where $\omega_{H}$ is cross-swing angular velocity, rad/s; $\omega_{r}$ is self-rotating angular velocity, rad/s.

Moreover, the cutting power and cutting specific energy consumption can be described:

7 $$P = \omega_{r} Mz/(10^{6} \eta )$$

8 $$H_{w} = P/3.6\lambda Av$$

where *P* is the cutting power, kW; *Mz* is the torque of cutting head, N·m; $\eta$ is the mechanical transfer efficiency; *H*_*w*_ is the cutting specific energy consumption, kW·h; $\lambda$ is the loose coefficient of coal-rock; *A* is the cross-sectional view of a cut made with a cutting head, m^2^.

## Structure design of cutting head

### Picks arrangement

According to the shape characteristics of the cutting head, the picks arrangement is designed in steps according to the cylindrical section, the conical section and the spherical section.

1. Cylindrical section

According to the cutting requirements of roadheader, the relationship between the intercept distance of the pick and the maximum cutting depth of the cutting head can be expressed as follows:

9 $$t_{1} = 2h_{\max } (\tan \phi - \tan \beta )$$

where *t*_1_ is the intercept distance, mm; *h*_max_ is the maximum cutting depth, mm; $\phi$ is the caving angle, °; $\beta$ is the spiral angle, °.

The number of intercept lines is:

10 $$N_{1} = floor[(L_{1} - L_{f} )/t_{1} + 1]$$

where *N*_1_ is the number of intercept lines on the cylindrical section; $floor$ is calculated by rounding down; *L*_1_ is the length of the cylindrical section, mm; *L*_*f*_ is the distance between the first intercept line and the large end face of the cutting head, mm.

Thus, the remaining length of the cylindrical section *L*_1*s*_ can be described as:

11 $$L_{1s} = L_{1} - L_{f} - t_{1} (N_{1} - 1)$$

The axial distance of picks from the large end face is derived as:

12 $$l_{z1} (i) = L_{f} + (i - 1) \cdot t_{1} ,\begin{array}{*{20}c} {} & {} & {i = 1,2,3, \ldots ,N_{1} } \\ \end{array}$$

where *l*_*z*1_(*i*) is the axial distance from the pick of the *i*-th intercept line on the cylindrical section to the large end face, mm.

The cutting radius of picks can be expressed as:

13 $$R_{1} (i) = \frac{D}{2}$$

where *D* is the diameter of cylindrical section, mm.

**Figure 3:**
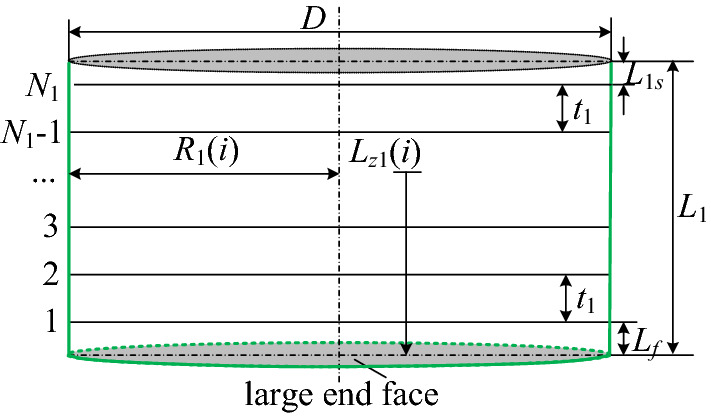
The design sketch of the cylindrical section.

The design sketch of the cylindrical section is shown in Fig. 3.

(2) Conical section

The intercept line arrangement of the conical section can be divided into two types: equidistance or equidifference, which are chosen according to the relationship between the remaining length of the cylindrical section and the intercept distance.

The number of intercept lines for each of the two types is:

Equidistance:

14 $$N_{2} = floor[(L_{2} + L_{1s} )/t_{2} ]$$

Equidifference:

15 $$N_{2} = \left( {1 - \sqrt {1 - 8floor(L_{1s} + L_{2} )/d} } \right)/2$$

where *N*_2_ is the number of intercept lines on the conical section; *L*_2_ is the length of the conical section, mm; *d* is the tolerance, *t*_2_ is the intercept distance of the conical section, mm.

Thus, the remaining length of the cylindrical section *L*_2*s*_ can be described as:

Equidistance:

16 $$L_{2s} = L_{1s} + L_{2} - (N_{2} - 1)t_{2}$$

Equidifference:

17 $$L_{2s} = L_{1s} + L_{2} - (t_{2} + N_{2} (N_{2} - 1)d/2)$$

The axial distance of the pick from the large end face is derived as:

Equidistance:

18 $$l_{z2} (i) = L_{1} - L_{1s} + (i - N_{1} ) \cdot t_{2} ,\quad i = N_{1} + 1,N_{1} + 2, \ldots ,N_{1} + N_{2}$$

Equidifference:

19 $$l_{z2} (i) = L_{f} + (N_{1} - 1) \cdot t_{1} + (i - N_{1} - 2) \cdot t_{2} - (i - N_{1} - 2) \cdot ((i - N_{1} - 2) - 1)d/2,\quad i = N_{1} + 1,N_{1} + 2, \ldots ,N_{1} + N_{2}$$

where *l*_*z*2_(*i*) is the axial distance from the pick of the *i*-th intercept line on the conical section to the large end face, mm.

The cutting radius of picks can be expressed as:

20 $$R_{2} (i) = \frac{D}{2} - \tan \psi \cdot (l_{z2} (i) - L_{1} )$$

where $\psi$ is the half cone angle of the conical section, °.

**Figure 4:**
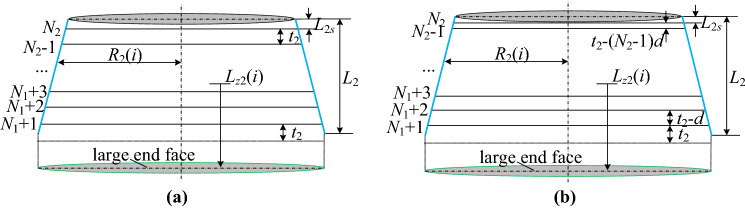
The design sketch of the conical section: (**a**) equidistance; (**b**) equidifference.

The design sketch of the conical section is shown in Fig. 4.

(3) Spherical section

There is a strong compression tension effect in the coal-rock crushing process. In order to avoid deformation or damage of the pick due to excessive force, the density of the pick at the spherical section should be gradually increased. Therefore, the pick arrangement of the spherical section adopts the equal degree principle, and the number of intercept lines is:

21 $$N_{3} = (90 - \phi )/\alpha$$

where *N*_3_ is the number of intercept lines on the spherical section; $\phi$ is the center angle corresponding to the first intercept line of the spherical section, °; $\alpha$ is the isometric principle angle, °.

The radius of the spherical section can be calculated by Eq. (22), and the cutting radius of picks can be expressed as Eq. (23).

22 $$R = \left( {\left( {\frac{D}{2} - L_{2} \cdot {\text{tan}}\psi } \right)^{2} + L_{3}^{2} } \right)/2L_{3}$$

23 $$R_{3} (i) = R\cos ((i - N_{1} - N_{2} - 1)\alpha + \phi )$$

where *R* is the radius of the spherical section, mm; *L*_3_ is the length of the spherical section, mm; *R*_3_(*i*) is the cutting radius of picks on the *i*-th intercept line, mm.

The axial distance of the pick from the large end face is derived as:

24 $$l_{z3} (i) = R_{3} \cdot \sin ((i - N_{1} - N_{2} - 1)\alpha + \phi ) - l_{f} + L_{1} + L_{2} ,\quad i = N_{1} + N_{2} + 1,N_{1} + N_{2} + 2, \ldots ,N_{1} + N_{2} + N_{3}$$

where *l*_*z*3_(*i*) is the axial distance from the pick of the *i*-th intercept line on the spherical section to the large end face, mm; *l*_*f*_ is the distance from the first intercept line of the spherical section to the spherical center, mm.

**Figure 5:**
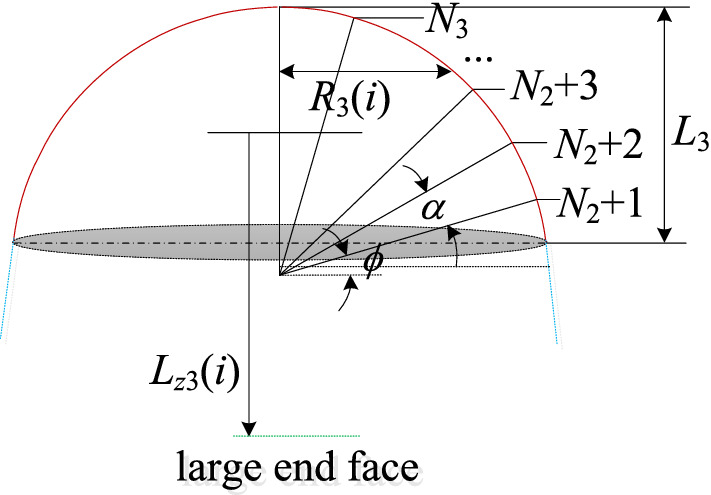
The design sketch of the spherical section.

The design sketch of the spherical section is shown in Fig. 5.

Ultimately, the total number of intercept lines *N* is determined:

25 $$N = N_{{1}} + N_{{2}} + N_{{3}}$$

The circumferential angle $\gamma_{b}$ is :

26 $$\gamma_{bi} = \frac{{360^\circ l_{z} (i)}}{{\pi D_{y} \tan \beta }},\quad i = 1,2,3, \ldots ,N$$

where *l*_*z*_(*i*) is the axial distance from the pick of the *i*-th intercept line to the large end face, mm; *D*_*y*_ is the outer edge diameter of helical vane, mm; $\beta$ is the spiral angle, °.

According to the theory of cutting head design, the relationship between the inclination angle of the picks and the deflection angle can be described as:

27 $$\left\{ {\begin{array}{*{20}l} {\varpi (i) = 8} \hfill & {\theta \le 80} \hfill \\ {\varpi (i) = 80 - \theta (i)} \hfill & {\theta > 80} \hfill \\ \end{array} } \right.$$

where $\varpi$ is the deflection angle, °; $\theta$ is the inclination angle, °.

Thus, the pick installation position can be determined by Eqs. (12), (13), (18), (19), (20), (24), (26), (27).

**Figure 6:**
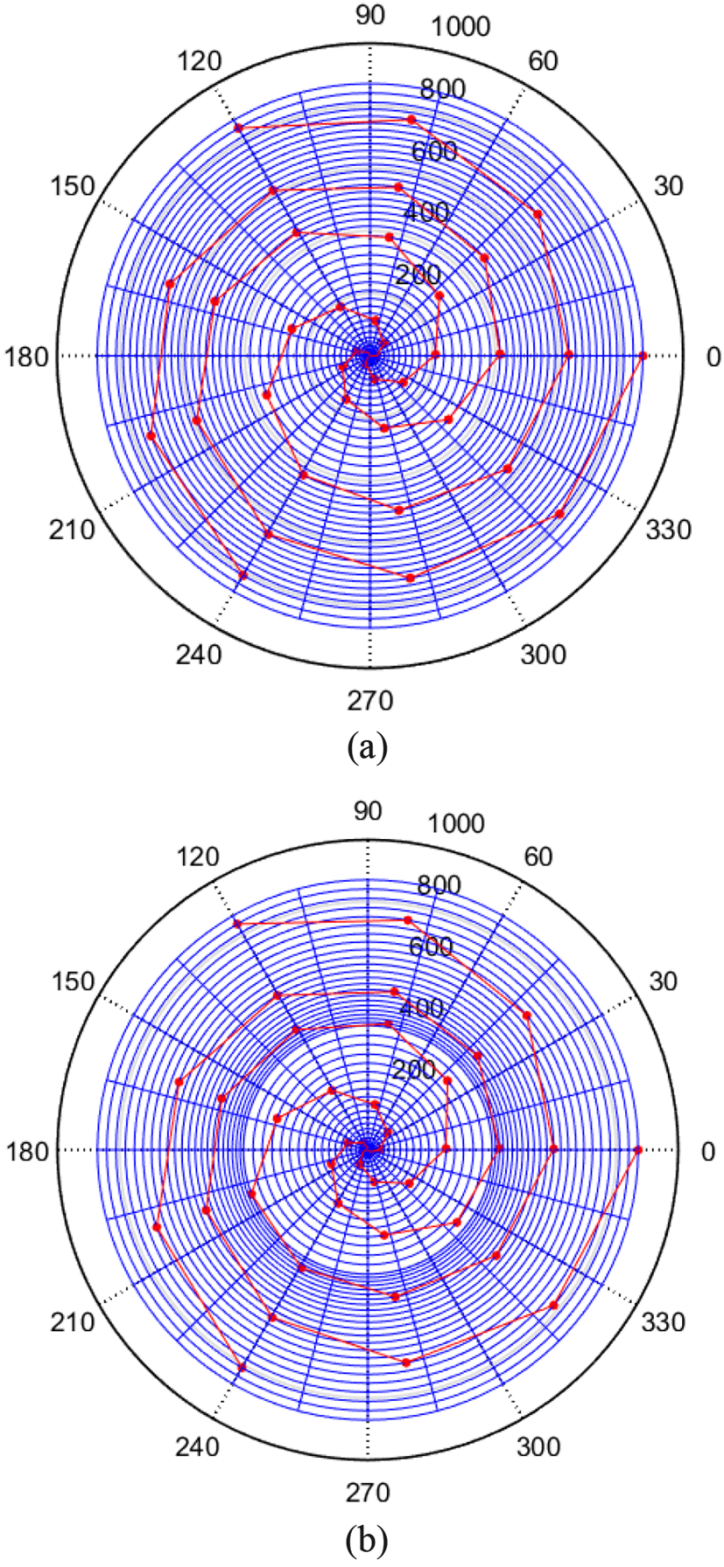
Pick arrangement diagram: (**a**) equidistance; (**b**) equidifference.

By compiling and running the program code, the pick arrangement diagrams under the above two types were obtained as shown in Fig. 6.

### Pick cutting diagram

**Figure 7:**
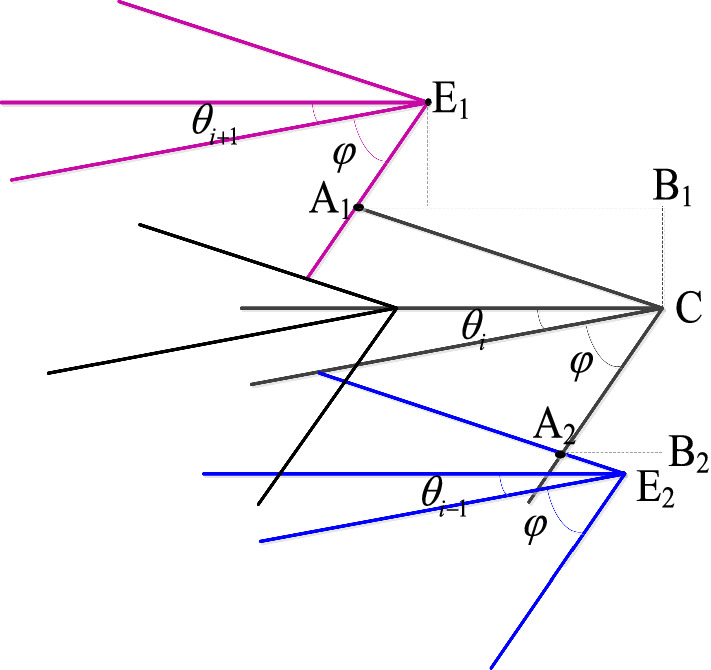
Schematic diagram of intersection point of the crumbling line.

The cutting diagram is an important basis for considering the size of the coal-rock. The larger the cutting area, the larger the rock lumpiness and the smaller the amount of dust, and the key to drawing the cutting diagram is to determine the upper and lower caving lines of the picks. The intersection point of the crumbling line of the pick on the *i*th intercept line during the cross-swing cutting of the sequential cutting head is shown in Fig. 7.

Where *E*_1_, *E*_2_ are the upper intersection point and lower intersection point respectively.

According to the geometric and kinematic relationship, the following equation can be obtained.

28 $$\left| {{\text{A}}_{{1}} {\text{B}}_{{1}} } \right| = \frac{{{\text{cot}}(\varphi - \theta_{i} ) \cdot (t\cot (\varphi + \theta_{i + 1} )) + (2\pi v_{i} /360\omega_{r} )}}{{\cot (\varphi - \theta_{i} ) + \cot (\varphi + \theta_{i + 1} )}}$$

29 $$\left| {{\text{A}}_{{2}} {\text{B}}_{{2}} } \right| = \frac{{{\text{cot}}(\varphi - \theta_{i - 1} ) \cdot (t\cot (\varphi + \theta_{i} )) + (2\pi v_{i} \Delta \gamma_{i - 1} /360\omega_{r} )}}{{\cot (\varphi - \theta_{i - 1} ) + \cot (\varphi + \theta_{i} )}}$$

The distance from the point *C* to the center axis of the cutting head is:

30 $$L_{c} = R_{Ti} + \frac{{2\pi v_{i} \gamma_{bi} }}{{360\omega_{r} }} + (S - 1)\frac{{v_{i} }}{m},\quad S = 1,2, \ldots$$

where the subscript *i* indicates the *i*-th intercept line; $\varphi$ is the caving angle, °; $\Delta \gamma_{i + 1}$, $\Delta \gamma_{{i{ - }1}}$ are the difference in circumferential angle of the picks on the adjacent intercept line; *R*_*Ti*_ is the cutting radius of picks on the *i*-th intercept line, mm; *m* is the number of helical vanes.

Therefore, the equations of the upper and lower caving lines can be described as:

31 $$y_{s} = \tan (\theta_{i} - \varphi )(x_{s} - L_{c} ) + l_{z} (i)\quad L_{c} - \left| {{\text{A}}_{{1}} {\text{B}}_{{1}} } \right| \le x_{s} \le L_{c}$$

32 $$y_{x} = \tan (\theta_{i} + \varphi )(x_{x} - L_{c} ) + l_{z} (i)\quad L_{c} - \left| {{\text{A}}_{{2}} {\text{B}}_{{2}} } \right| \le x_{x} \le L_{c}$$

**Figure 8:**
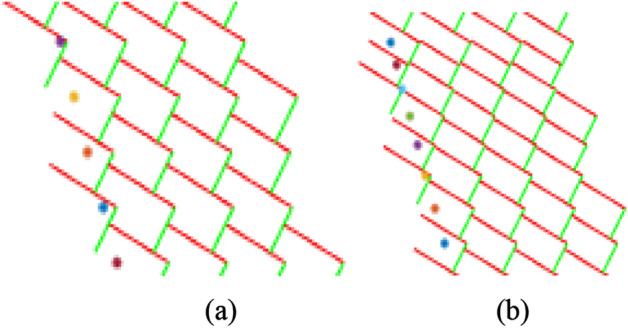
Cutting diagram: (**a**) equidistance; (**b**) equidifference.

By compiling and running the program code, the cutting diagrams under the above two types were obtained as shown in Fig. 8.

## Construction and verification of virtual prototype model

### Construction of coupling model

**Figure 9:**
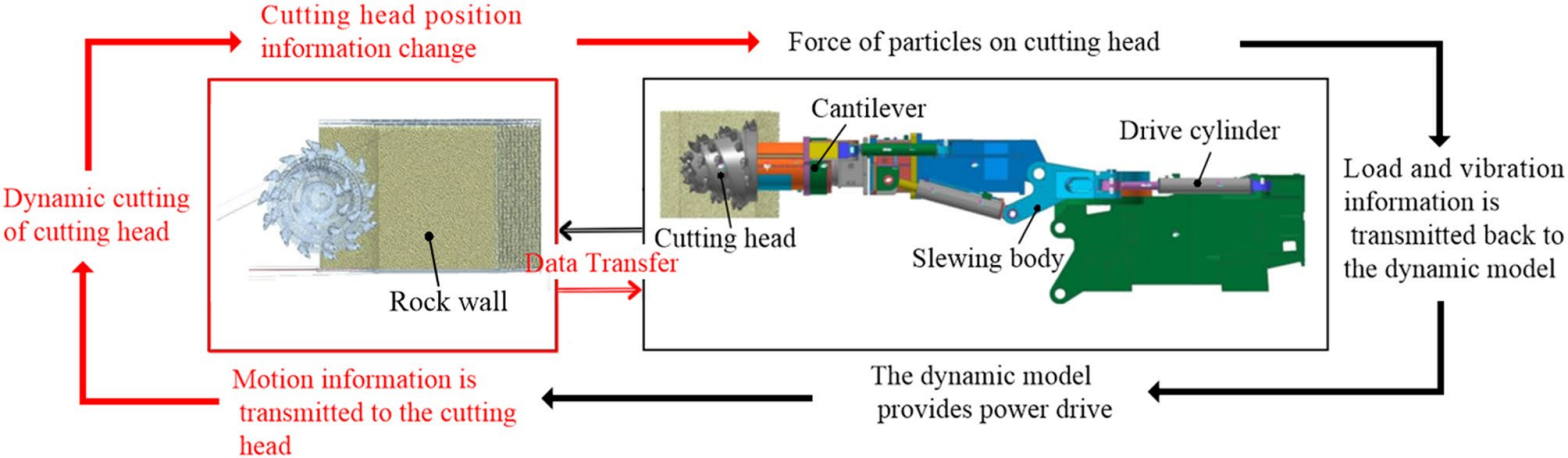
Coupling model and data transfer principle.

In this study, the cutting performance information is obtained based on the virtual prototype technology. Firstly, a 3D model of the cutting mechanism of the roadheader is established using Pro engineer, and an arc-shaped rock wall model with the slewing mechanism as the center and the cutting arm as the radius is established according to the cross-swing trajectory of the roadheader. Secondly, the rock wall model is imported into the discrete element software for particle filling and bonding to generate a discrete element model of the rock wall. Then, the 3D model of the cutting mechanism is imported into the multi-body dynamics software. According to the working principle of the cutting mechanism, constraints and drives are added to the parts to establish the dynamics model of the cutting mechanism. Finally, the cutting head is used as the coupling part with the rock wall to establish the coupling model. The coupling model and data transfer principle are shown in Fig. 9^40^.

**Figure 10:**
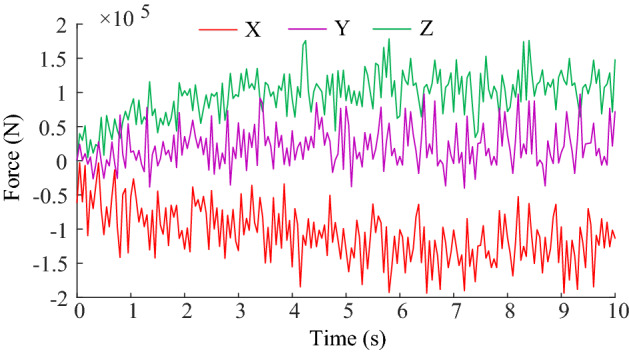
Load curves of the cutting head.

Through simulation, the load curves of the cutting head is obtained as shown in Fig. 10.

From the Fig. 10, it can be seen that in the cutting process, the load in X, Y, Z direction of the cutting head presents nonlinearity and fluctuation. The peak force in the X-direction is 1.9369 × 10^5^ N, the peak force in the Y-direction is 9.7436 × 10^4^ N and the peak force in the Z-direction is 1.7826 × 10^5^ N. The average force in X-direction is 1.0217 × 10^5^ N, the average force in Y-direction is 1.9670 × 10^4^ N, the average force in Z-direction is 9.0772 × 10^4^ N. This means that the load amplitude in the vertical direction is larger and the load amplitude in the cutting depth direction is relatively small.

Based on the load data obtained from simulation, the cutting power and cutting specific energy consumption can be calculated according to Eqs. (7) ~ (8).

### Experimental validation

**Figure 11:**
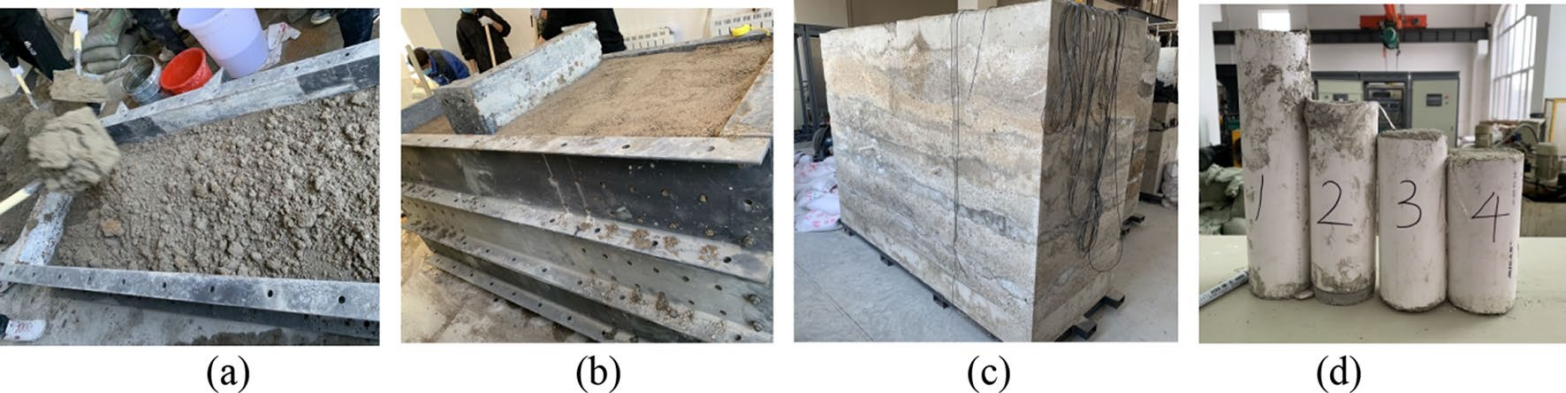
Rock wall construction and specimens: (**a**) filling; (**b**) compacting; (**c**) rock wall model; (**d**) specimens.

**Figure 12:**
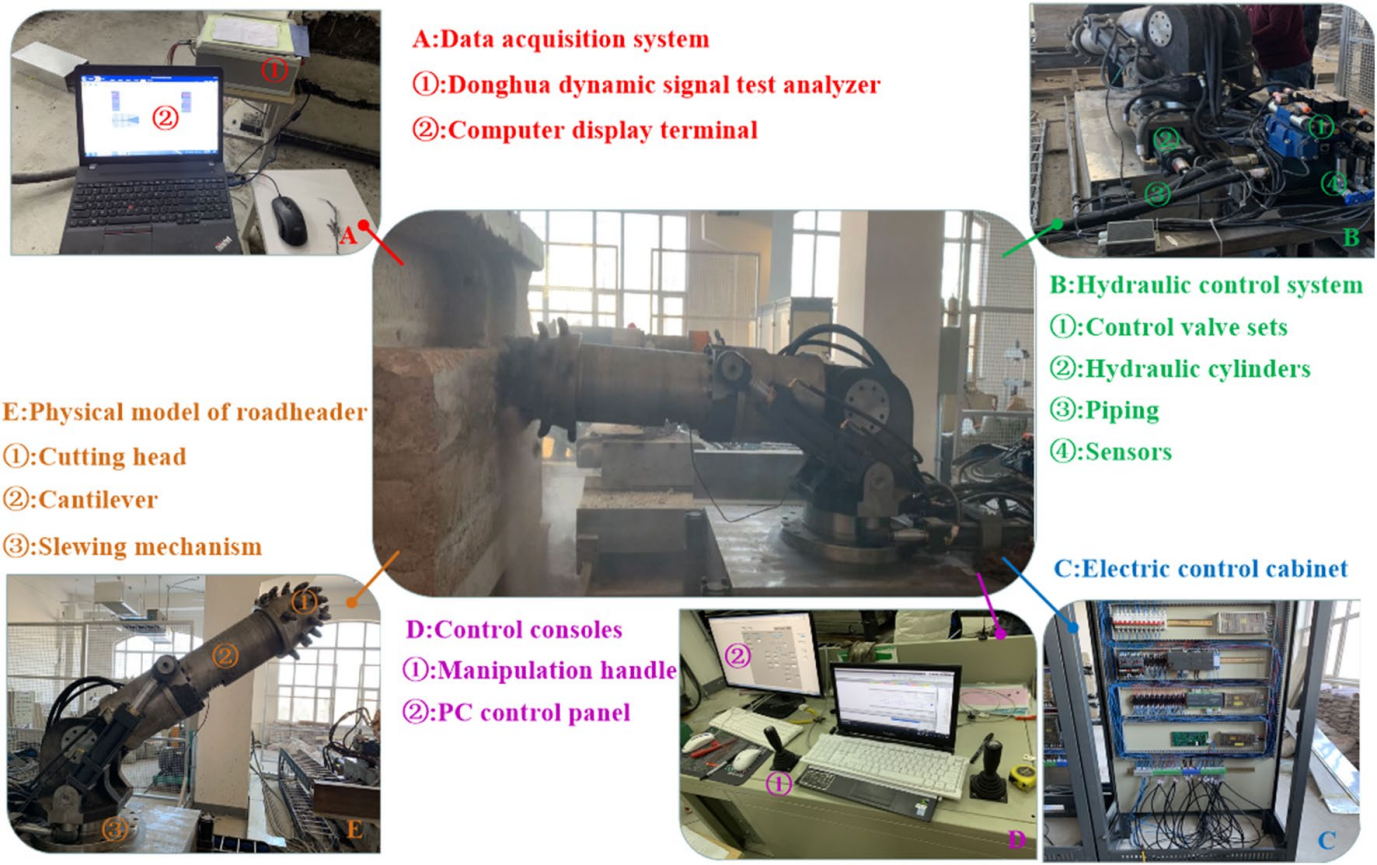
Cutting test bench.

In order to verify the accuracy of the load information obtained based on virtual simulation, an cutting test bench for cutting head was built. Firstly, the physical experiment model of rock wall was established, and the similarity between the rock wall model and the actual rock wall downhole was proved by uniaxial compression test on the rock wall specimens (Fig. 11). Secondly, a physical model of roadheader, the hydraulic control system, the electric control cabinet and the control consoles were built. Then, the cutting head is controlled by the computer control platform to cut the rock wall, and the load information of cutting head is obtained through the data acquisition system (Fig. 12).

**Figure 13:**
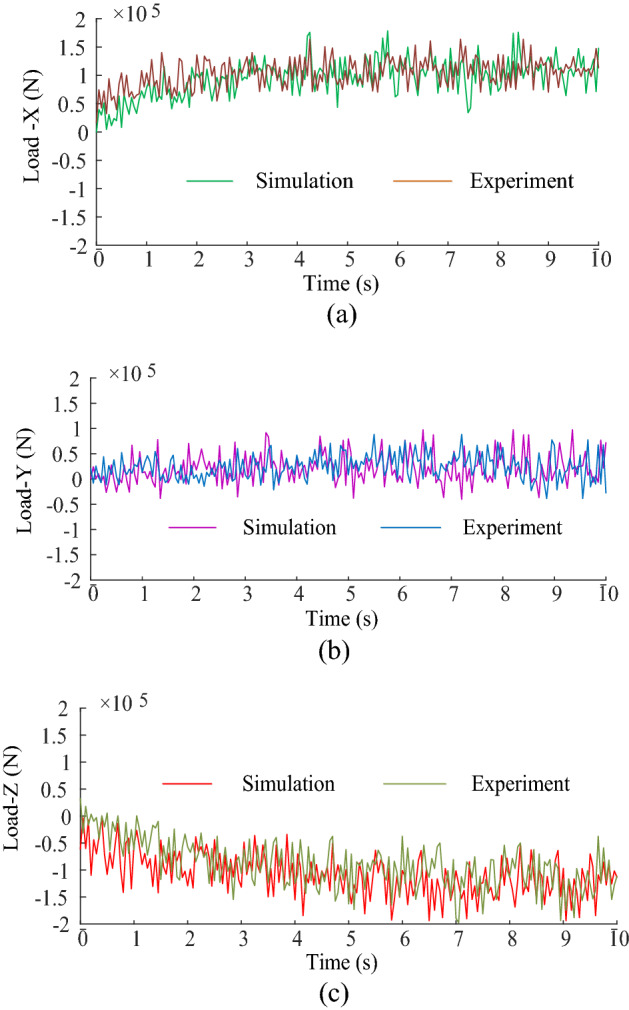
The comparison curves between experimental force and simulation result: (**a**) X-direction; (**b**) Y-direction; (**c**) Z-direction.

Figure 13 shows the comparison curves between experimental load and simulation result of the cutting head. From Fig. 13, it can be seen that the simulated and experimental curves have similar fluctuation and overall change trend. Based on the calculations, it can be known that the simulation and experimental results have an error of 8% for the peak load in the X-direction, 9% for the peak load in the Y-direction and 2% for the peak load in the Z-direction, and that the error for the mean load in the X-direction is 8%, the error for the mean load in the Y-direction is 18% and the error for the mean load in the Z-direction is 16%. Accordingly, we can conclude that it is feasible to obtain the load information of the cutting head based on the virtual prototype technology. Therefore, the virtual simulation can be used to obtain the load information of the cutting head under different working conditions, and then obtain the cutting performance. And the advantages of virtual simulation can be fully utilized in the design of cutting heads to simplify the design process, shorten the design cycle and reduce design costs.

## Cutting performance of the cutting head

By performing a large number of simulations, the relationship surfaces for cutting performance under different parameters are constructed. The following is the examples of the isometric principle angle, spiral angle, cross-swing speed and rotation speed.

### Isometric principle angle and spiral angle

**Figure 14:**
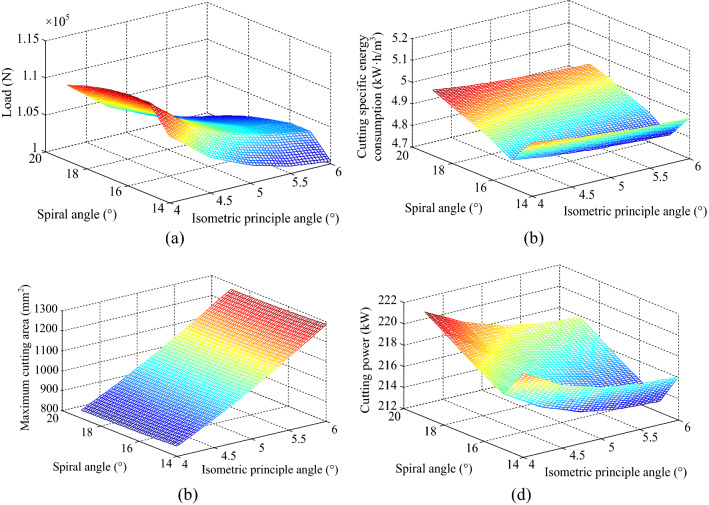
Relationship surfaces of each cutting performance index with the isometric principle angles and spiral angles: (**a**) cutting load; (**b**) cutting specific energy consumption; (**c**) maximum cutting area; (**d**) cutting power.

The cutting head with the isometric principle angles of 4°, 4.5°, 5°, 5.5° and 6° and the spiral angles of 14°, 16°, 18° and 20° were established respectively, and the cutting load, cutting specific energy consumption, maximum cutting area and cutting power were obtained by simulation and calculation, and the relationship surfaces of each cutting performance index with the isometric principle angle and spiral angle were fitted, as shown in Fig. 14.

From Fig. 14a, it can be seen that with the increase of spiral angle, the cutting load increases and then decreases; with the increase of isometric principle angle, the cutting load has a non-linear decreasing trend.

From Fig. 14b and d, it can be seen that with the increase of spiral angle, the cutting specific energy consumption and cutting power of cutting head first decreases and then increases. When the spiral angle is 15.5°, both of them reach the minimum value; with the increase of the isometric principle angle, the cutting specific energy consumption and cutting power show a non-linear and gentle decreasing trend.

From Fig. 14c, it can be seen that with the increase of the spiral angle, the maximum cutting area decreases. This is due to the fact that the increase of the spiral angle can reduce the distance between the picks on the two intercept lines in the circumferential direction, thus shortening the length of the upper and lower caving lines, resulting in a smaller cutting area; with the increase of the isometric principle angle, the maximum cutting area shows an approximately linear increasing trend. The effect of the angle of the isometric principle on the maximum cutting area is more significant.

### Cross-swing speed and rotation speed

**Figure 15:**
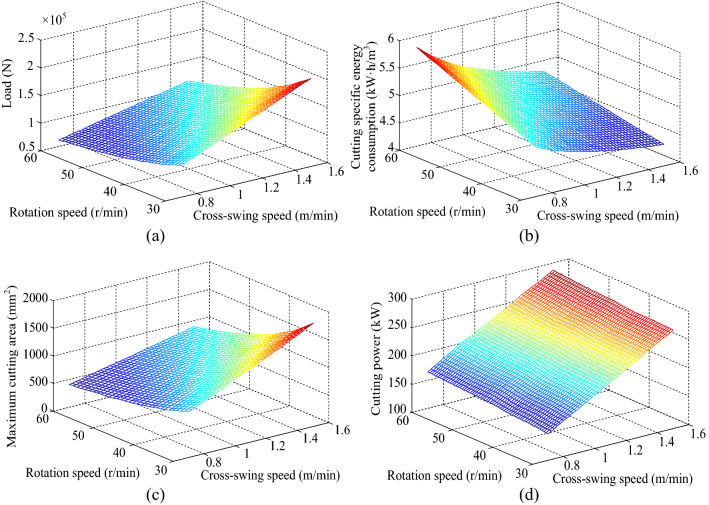
Relationship surfaces of each cutting performance index with the cross-swing speeds and rotation speeds: (**a**) cutting load; (**b**) cutting specific energy consumption; (**c**) maximum cutting area; (**d**) cutting power.

By adjusting the cross-swing speed and rotation speed of cutting head, the cutting load, cutting specific energy consumption, maximum cutting area and cutting power were obtained by simulation and calculation, and the relationship surfaces of each cutting performance index with the cross-swing speed and rotation speed were fitted, as shown in Fig. 15.

From Fig. 15a,c, it can be seen that the maximum cutting area and cutting load increase with the increase of the cross-swing speed and decrease with the increase of the rotation speed. This is due to the fact that increasing the cross-swing speed can increase the cutting distance of the picks per unit time, resulting in the increase of the cutting area. When the volume of rock material to be cut is the same, the higher the rotation speed, the shorter the cutting cycle of single pick, and the more cutting times, which leads to the reduction of cutting area.

From Fig. 15b, it can be seen that the cutting specific energy consumption is non-linearly decreasing with the increase of the cross-swing speed, and the change rate is gradually decreasing; the cutting specific energy consumption is non-linearly increasing with the increase of the rotation speed. The effect of the cross-swing speed on the cutting specific energy consumption is more significant.

From Fig. 15d, it can be seen that the increase of the cross-swing speed and the rotation speed can increase the cutting power in a nearly linear trend, and the influence of the cross-swing speed on the cutting power is more obvious. This is because increasing the cross-swing speed can increase the cutting distance of the picks, and once the cutting distance increases, the power consumed by the cutting motor will increase; while the rotation speed determines the number of picks involved in cutting per unit time, and the more the number of picks, the higher the cutting power required.

Cutting performance data under other parameters are obtained, so that a database matching cutting performance with structural parameters, working parameters and coal-rock properties is established for performance optimization in the cutting head design process.

**Figure 16:**
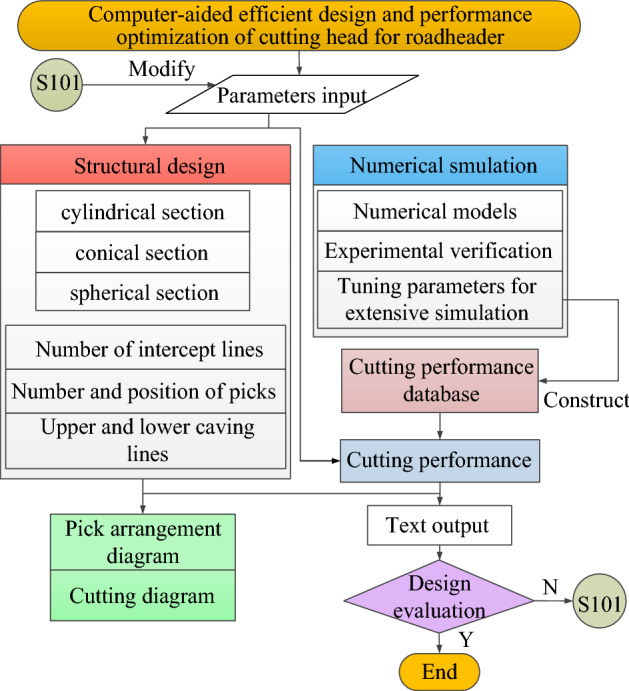
The flow of computer-aided efficient design and performance optimization of the cutting head for roadheader.

**Figure 17:**
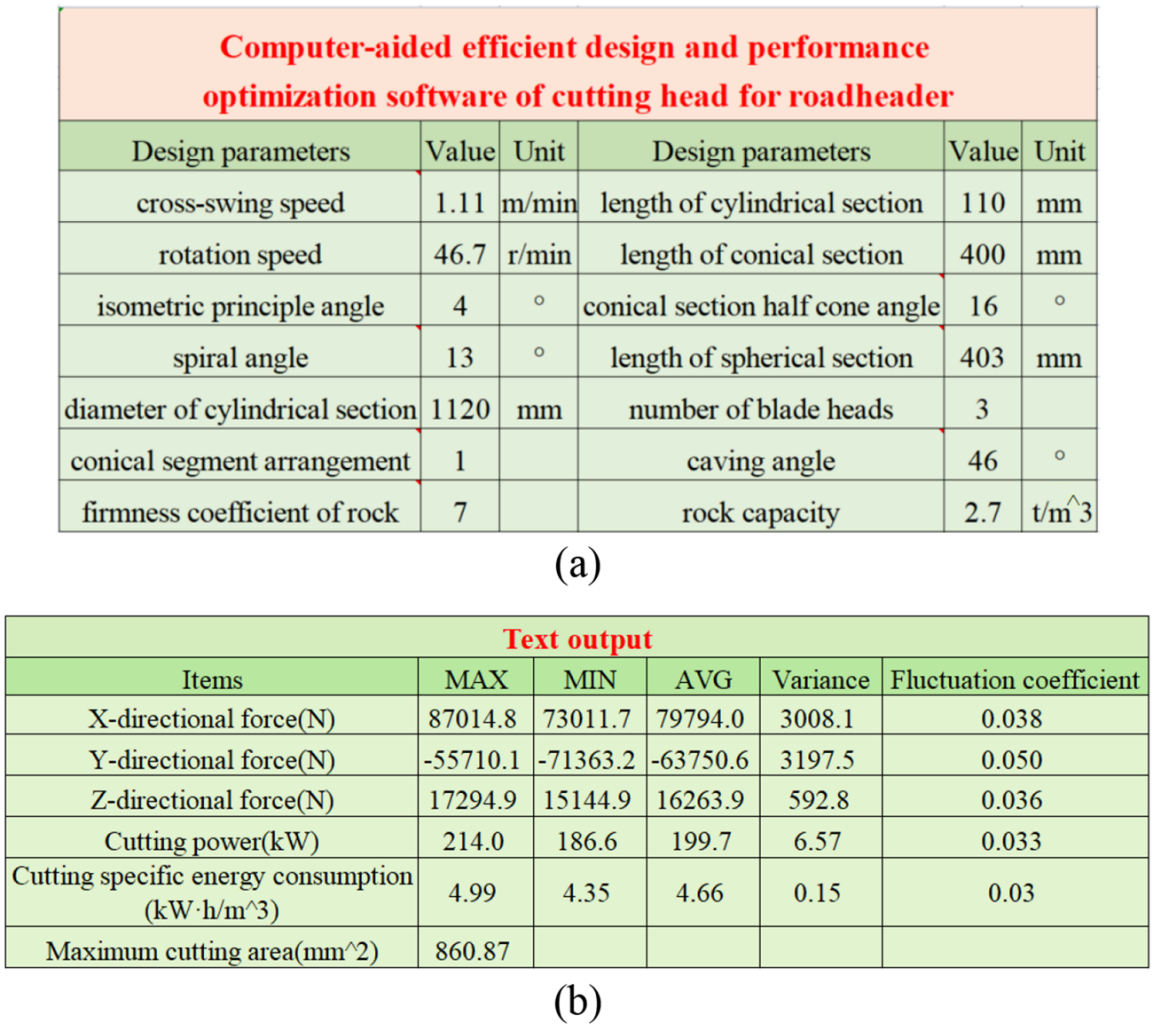
Parts of the interface and program code: (**a**) partial parameter input interface; (**b**) partial text output interface.

The flow of computer-aided efficient design and performance optimization of the cutting head for roadheader is shown in Fig. 16. During the design process, the design parameters need to be input into EXCEL, and then the program code is executed to read the relevant parameters through “**xlsread**” to calculate the corresponding pick arrangement and cutting performance data, and finally stored in EXCEL. If the design result is not satisfactory, the design parameters can be adjusted to modify the structure of the cutting head to achieve the purpose of optimizing the performance. Parts of the interface and program code are shown in Fig. 17.

It should be noted that the spiral angle is both a design parameter for the pick arrangement and an influencing factor for the cutting performance of cutting head. Furthermore, in the case of a longitudinal cutting head, the spiral angle is equivalent to the angle of the helical vanes responsible for moving the output towards the roadheader’s loader. It has a significant impact on the output loading effect, so its influence should therefore be considered in advance and a reasonable range set for the spiral angle to meet the output loading capacity when carrying out computer-aided optimization design.

**Figure 18:**
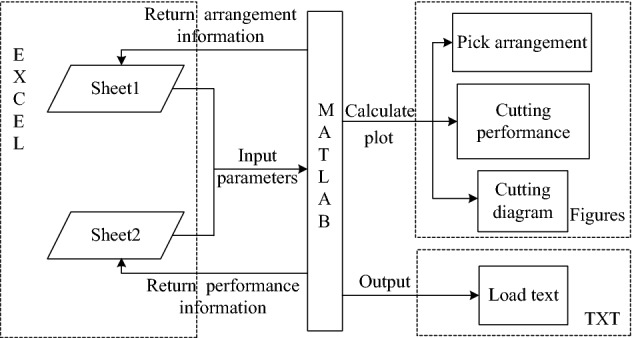
The schematic diagram of how this works.

In summary, the computer-aided efficient design and performance optimization software mainly implements the joint calculation of EXCEL and MATLAB, where EXCEL is responsible for inputting basic design parameters, cutting parameters, etc., and MATLAB is responsible for calculating and returning the results, including pick installation information, performance information, and generating figures and load text, which can be used for dynamics analysis, reliability analysis. The schematic diagram of how this works is shown in Fig. 18.

## Engineering applications

We designed the EBZ220 roadheader cutting head by the computer-aided structure design and performance optimization method, and obtained the optimal design scheme and performance data as shown in Table 1.

**Table 1:** Optimal scheme for the design of the cutting head.

Projects	Value
Cross-swing speed (m/min)	1.106
Rotation speed (r/min)	46.7
Spiral angle (°)	15.5
Isometric principle angle (°)	4
Cutting specific energy consumption (kW·h/m^3^)	4.9
Maximum cutting area (mm^2^)	854.95
Cutting power (kW)	217.49
Cutting load (kN)	112.20

**Figure 19:**
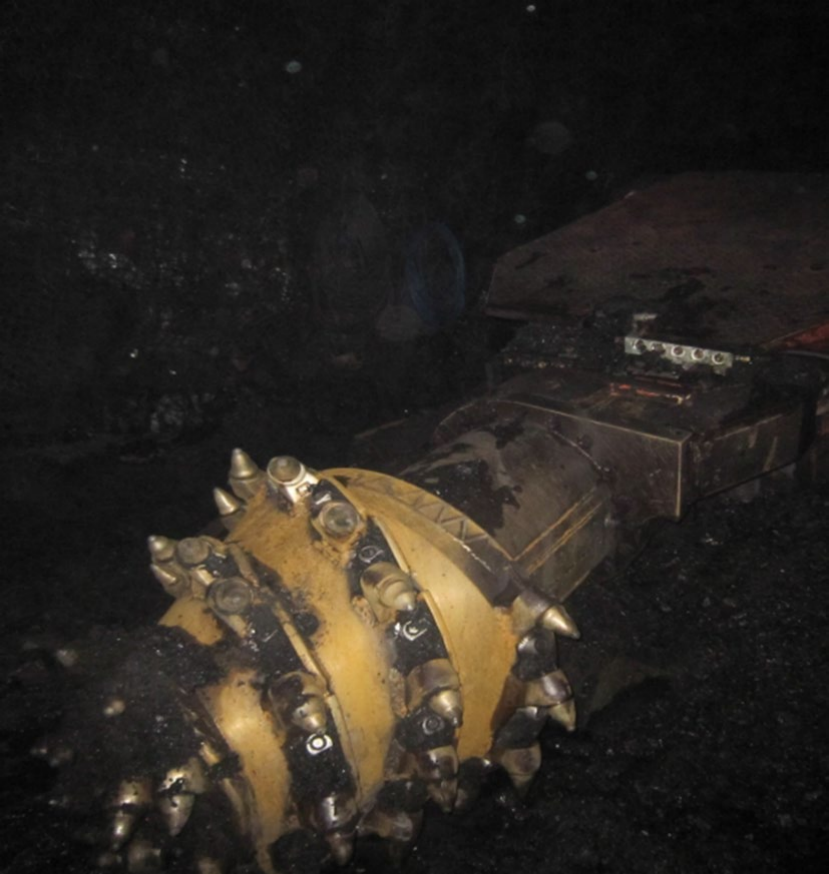
Cutting head of EBZ220 roadheader in underground mining.

The cutting head was designed and manufactured according to the structural parameters in Table 1 and used in downhole mining (Fig. 19). Through long-term monitoring of the working stability and cutting performance of the roadheader, we find that the cutting head no obvious breakage phenomenon and is highly reliable. The production goals of high efficiency, economy and quality are achieved, which shows that the proposed approach is feasible.

## Conclusion

We present a method for the structure design and performance optimization of cutting head. The core components of the proposed approach include compiling the calculation code jointly with EXCEL and obtaining the cutting head load information and other performance data based on the virtual prototype technology. The establishment and simulation of the coupling model of the cutting head cutting rock wall plays a crucial role for the accuracy and effectiveness of the proposed approach. The coupled model of cutting head cutting rock wall is verified by physical experiments. The cutting head designed by the proposed approach in this paper was also validated by downhole mining tests, which showed that the cutting head created by the proposed approach works reliably and has high performance. For future work, if the wear characteristics can be considered in the performance optimization of the cutting head, it is significant to extend its service life and improve the economic efficiency.
